# Type I interferons and related pathways in cell senescence

**DOI:** 10.1111/acel.13234

**Published:** 2020-09-12

**Authors:** Steven M. Frisch, Ian P. MacFawn

**Affiliations:** ^1^ Department of Biochemistry and WVU Cancer Institute West Virginia University Morgantown West Virginia USA; ^2^ Present address: Department of Immunology, Hillman Cancer Center University of Pittsburgh Pittsburgh Pennsylvania USA

**Keywords:** cellular immunology, cellular senescence, DNA damage, inflammation, longevity, senescence

## Abstract

This review article addresses the largely unanticipated convergence of two landmark discoveries. The first is the discovery of interferons, critical signaling molecules for all aspects of both innate and adaptive immunity, discovered originally by Isaacs and Lindenmann at the National Institute for Medical Research, London, in 1957 (*Proceedings of the Royal Society of London. Series B: Biological Sciences*, 1957, **147**, 258). The second, formerly unrelated discovery, by Leonard Hayflick and Paul Moorhead (Wistar Institute, Philadelphia) is that cultured cells undergo an irreversible but viable growth arrest, termed senescence, after a finite and predictable number of cell divisions (*Experimental Cell Research*, 1961, **25**, 585). This phenomenon was suspected to relate to organismal aging, which was confirmed subsequently (*Nature*, 2011, **479**, 232). Cell senescence has broad‐ranging implications for normal homeostasis, including immunity, and for diverse disease states, including cancer progression and response to therapy (*Nature Medicine*, 2015, 21, 1424; *Cell*, 2019, **179**, 813; *Cell*, 2017, 169, 1000; *Trends in Cell Biology*, 2018, **28**, 436; *Journal of Cell Biology*, 2018, **217**, 65). Here, we critically address the bidirectional interplay between interferons (focusing on type I) and cell senescence, with important implications for health and healthspan.

## Graphical abstract

Interferon I (IFN‐I) expression is important not only for amplifying immune responses but also for enforcing cell senescence. Pattern recognition receptor activation, DNA damage signaling, and retrotransposition can trigger senescence through IFN‐I, and, reciprocally, IFN‐I can stimulate DNA damage signaling. Mechanisms are discussed in this review.

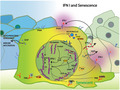

**Abbreviations**
APC: antigen‐presenting cell
ATM: ATM serine/threonine kinase
CCFs: cytosolic chromatin fragments
cGAS: cyclic GMP‐AMP synthase
CHK: checkpoint kinase
CSC: cancer stem cell
DAMPs: damage‐associated molecular patterns
DC: dendritic cell
DDR: DNA damage response
EMT: epithelial–mesenchymal transition
GM‐CSF: granulocyte–macrophage colony‐stimulating factor
IDO: indoleamine 2,3‐dioxygenase
IFN: interferon
IFNAR1: interferon alpha receptor 1
IL‐1,‐6: interleukin‐1,‐6
IRF: interferon regulatory factor
ISGF3: interferon‐stimulated gene factor 3
MDA5: melanoma differentiation‐associated protein 5
MET: mesenchymal–epithelial transition
NF‐kB: nuclear factor kappa‐light‐chain‐enhancer
NK: natural killer cell
OIS: oncogene‐induced senescence
PAMPs: pathogen‐associated molecular patterns
PARP: poly‐ADP ribose polymerase03b
PRRs: pattern recognition receptors
Rb: retinoblastoma protein
RIG1: retinoic acid‐inducible gene 1
RLR: RIG‐I‐like receptor
ROS: reactive oxygen species
SASP: senescence‐associated secretory phenotype
SCARS: DNA segments with chromatin alterations reinforcing senescence
STAT: signal transducer and activator of transcription
STING: stimulator of interferon genes
TGF‐β: transforming growth factor beta
TLR: Toll‐like receptor
VEGF: vascular endothelial growth factor
γ‐H2AX: 140S phosphorylated H2A histone family member X

“If there's one lesson I've learned from studying the immune system, it is that evolution doesn't select for happiness”. Matthias Wabl (UCSF)

## 1 NEW FACETS OF INTERFERON‐IS STILL REMAIN TO BE DISCOVERED

Type I interferons are encoded by the thirteen human IFN‐α genes, the single IFN‐β gene, and by additional genes of the IFN‐ε, IFN‐κ and IFN‐o families, and are expressed ubiquitously. Epithelial cells additionally express type III interferons (IFN‐λs), especially at barrier tissues where a localized, non‐systemic response is advantageous. A specialized innate immune cell called plasmacytoid dendritic cells (pDCs) secrete large amounts of IFN‐I in the setting of viral infections, but tend to be inactivated by tumors or persistent viral infections (Snell, McGaha, & Brooks, 2017; Zuniga, Liou, Mack, Mendoza, & Oldstone, 2008). Pattern recognition receptors (PRRs)—the sensors of innate immunity—induce the type I and type III genes rapidly. These receptors recognize molecules presented by pathogens (pathogen‐associated molecular patterns, PAMPs), such as double‐stranded RNA (dsRNA), bacterial lipopolysaccharides, flagellin, bacterial lipoproteins, and cytosolic DNA (Amarante‐Mendes et al., 2018). Additional PRRs recognize molecules from damaged cells called damage/danger‐associated molecular patterns (DAMPs), notably including extracellularly derived cytosolic DNA, S100 proteins, high‐mobility group box 1 (HMGB1), and heat shock proteins (Roh & Sohn, 2018). Lower endogenous levels of dsRNA or cytosolic chromatin fragments, or perhaps other pseudo‐PAMPs/DAMPs, do occur—avoidance of self‐recognition is imperfect here—and are capable of stimulating their corresponding PRRs, with important ramifications. In addition to these “conventional” IFN‐I stimuli, other stimuli have more recently been revealed, including FAS/FASL activation (Qadir, Stults, Murmann, & Peter, 2020), DNA damage, endogenous cytosolic chromatin fragments, or dsRNA, with important implications for both senescence and efficacy of cancer therapy—(discussed here) (Deng et al., 2014; Sistigu et al., 2014; Yu, Katlinskaya, et al., 2015).

The signaling mechanisms leading from PRRs to IFN‐I and IFN‐III induction have been reviewed in detail elsewhere (Ablasser & Hur, 2020; Aleynick, Svensson‐Arvelund, Flowers, Marabelle, & Brody, 2019; Lukhele, Boukhaled, & Brooks, 2019). Briefly, for example, dsRNA is sensed by cytosolic receptors *RIG1* and *MDA5* as well as the endosomal Toll‐like receptor, TLR3. Cytosolic DNA is sensed by TLR9 and cGAS; the latter synthesizes an alarmin, cyclic GMP‐AMP (cGAMP), that stimulates STING, a central activator of IFN‐I induction. Primarily, signals from pattern recognition receptors converge upon IKK‐family kinases to phosphorylate and activate two transcription factors, IRF3 and NF‐kB; these factors directly transactivate the IFN‐β gene and, in epithelial cells, IFN‐λ genes as well as certain antiviral target genes. These early response (IRF3‐dependent but IFN‐I‐independent) genes include ISG15, a ubiquitin‐like peptide that regulates protein function by conjugation (Villarroya‐Beltri, Guerra, & Sanchez‐Madrid, 2017), and some antiviral genes including IFIT‐ and IFITM‐family genes, and MxA/B. Interestingly, STAT1 is also induced (Ashley, Abendroth, McSharry, & Slobedman, 2019). IFN‐β resulting from this first wave activates the IFNAR1/2 receptors, resulting in Janus kinase‐mediated phosphorylation of STAT1 and STAT2, forming a canonical complex of STAT1/STAT2/IRF9, known as the ISGF3 complex, that transactivates target genes (interferon‐stimulated genes, ISGs) containing ISRE (ISGF3 response elements); their unique characteristics have been reviewed in Ng, Mendoza, Garcia, and Oldstone (2016). These include numerous cytokines, the RIG1 and MDA5 genes themselves, and several IRFs, one of which, IRF7, drives a second wave of IFN‐β expression. A battery of antiviral genes is induced, which interfere with intracellular vesicle trafficking, and the stability and translation of viral mRNAs. Simultaneously, autocrine IFN‐I or paracrine IFN‐γ (IFN‐II, derived from NK or T‐helper cells) engages p53 and Rb checkpoint pathways that arrest growth (Braumuller et al., 2013; Sangfelt et al., 1999). Not surprisingly, this p53 activation is biologically indistinguishable from a DNA damage response (DDR), with important biological consequences, addressed below (Moiseeva, Mallette, Mukhopadhyay, Moores, & Ferbeyre, 2006). Growth arrest can also result from repression of c‐myc through IFN‐I induction of IFI16 (a cytosolic DNA sensor) or interference with transactivation by *MYC* and *E2F* proteins via the IFI202a/b gene products (Song et al., 2010). In certain contexts, an alternative and surprisingly efficient antiviral mechanism—apoptosis—is mediated by poly‐ubiquitinated IRF3 in a process termed RLR‐induced IRF‐3‐mediated apoptosis (RIPA) (Chattopadhyay, Kuzmanovic, Zhang, Wetzel, & Sen, 2016).

The critical roles of interferons in innate and adaptive immune responses, which have been reviewed elsewhere, neither can be overestimated in the settings of infection nor tumor surveillance. Briefly, acutely acting IFN‐Is induce dendritic cell maturation/MHC‐II expression, enhance cross‐presentation, stimulate the activity and/or proliferation of CD8, Th1, and Tfh cells, suppress T_reg_ differentiation, stimulate monocyte recruitment (e.g., via CCL2), and activate NK cell cytolytic activity. Nevertheless, chronic interferon stimulation is immunosuppressive in tumor microenvironments through the induction of PD1, Tim‐3, LAG‐3 checkpoint receptors on T cells, PD‐L1, and IDO on target cells and APCs, down‐regulation of IFN‐γ receptor on macrophages, dampening of DC expansion, and, paradoxically, suppression of T‐cell expansion (Benci et al., 2016; Lee & Ashkar, 2018). Note that this immune cell exhaustion—which is reversible—does not fit the definition of senescence, although it may be related. Nevertheless, IFN‐Is (and IFN‐γ) clearly represent double‐edged swords: They are acutely beneficial but chronically detrimental (Lee & Ashkar, 2018; Lukhele et al., 2019; Snell et al., 2017). The dual nature of IFN‐Is is also reflected in its effects on senescence in fibroblasts and epithelial cells, discussed herein, as senescence is itself contextually double‐edged.

Finally, it should be noted that various suppressors of IFN‐I expression or signaling have emerged, with physiologic relevance to immunity and perhaps also senescence. These include the aryl hydrocarbon receptor (AHR), the progesterone receptor (PR), and, with particular relevance to macrophage‐rich tumor microenvironments, tumor‐associated macrophages (TAMs) (Salvagno et al., 2019; Walter et al., 2017; Yamada et al., 2016).

## 2 CELL SENESCENCE, REVISITED

The explanation for the Hayflick limit *in fibroblasts* mentioned in our introduction is fairly straightforward, but it has profound implications for our understanding of senescence in general, which is not. Normal fibroblasts in culture undergo a finite number of divisions that are marked by progressive telomere shortening. Failure to maintain telomeres causes extreme genomic instability with persistent DNA damage repair (DDR) signaling. DDR, in turn, activates p53‐ and Rb‐dependent checkpoints, via cell cycle inhibitors including p16^INK4a^, p14ARF, and p21^WAF/CIP^. Temporary growth arrest can transition to permanence (>3 years has been observed) via (a) epigenetic modifications, primarily H3 K9 histone methylation followed by demethylation, that lead to a state of epigenetic chaos or noise (Kane & Sinclair, 2019; Sidler, Kovalchuk, & Kovalchuk, 2017; Takahashi et al., 2012), (b) stable, irreparable “DNA Segments with Chromatin Alterations Reinforcing Senescence” (SCARS) or damaged telomeres (Fumagalli et al., 2012; Rodier et al., 2011), and (c) the autocrine expression of senescence‐stabilizing factors. These factors, initially identified as matrix metalloproteinases, interleukin‐1 and interferon‐inducible genes, were later found to comprise a program generically called the senescence‐associated secretory program (SASP) (Bauer, Kronberger, Stricklin, Smith, & Holbrook, 1985; Maier, Voulalas, Roeder, & Maciag, 1990; Rodier et al., 2009; Tahara et al., 1995; West, Pereira‐Smith, & Smith, 1989). SASP compositions are notably variable across cell types, but most are targets of NF‐κB, which is activated by DDR and by SASP cytokines, for example, IL‐6. The astonishing longevity of senescent fibroblasts may relate to the up‐regulated expression of pro‐survival genes such as Bcl‐xl and Bcl‐2, downstream of NF‐κB. The transcription factor CEBP‐β also participates in activating the SASP, as do core signaling molecules including mTOR/IL1/IL1R/NF‐κB and the stress kinase, p38 MAPK (Laberge et al., 2015). Certain SASP components confer senescence upon neighboring cells, including IGFBP‐7, TGF‐β GRO‐α and, importantly, IFN‐Is (Gorgoulis et al., 2019). The IL‐6‐related cytokine, oncostatin‐M (OSM), for example, induces senescence through a STAT3‐mediated activation of the TGF‐β/Smad3 pathway (Bryson, Junk, Cipriano, & Jackson, 2017). Other SASP components, such as IL‐6, IL‐1, IL‐8, TNF, CXCL/CCL chemokines, matrix proteases, and growth factors (VEGF, GM‐CSF), promote a pro‐inflammatory environment that presumably aids in macrophage‐, T‐cell‐, or NK cell‐mediated clearance of senescent cells—an important tumor suppressor mechanism in the stromal microenvironment (Gluck & Ablasser, 2019).

**FIGURE 1:**
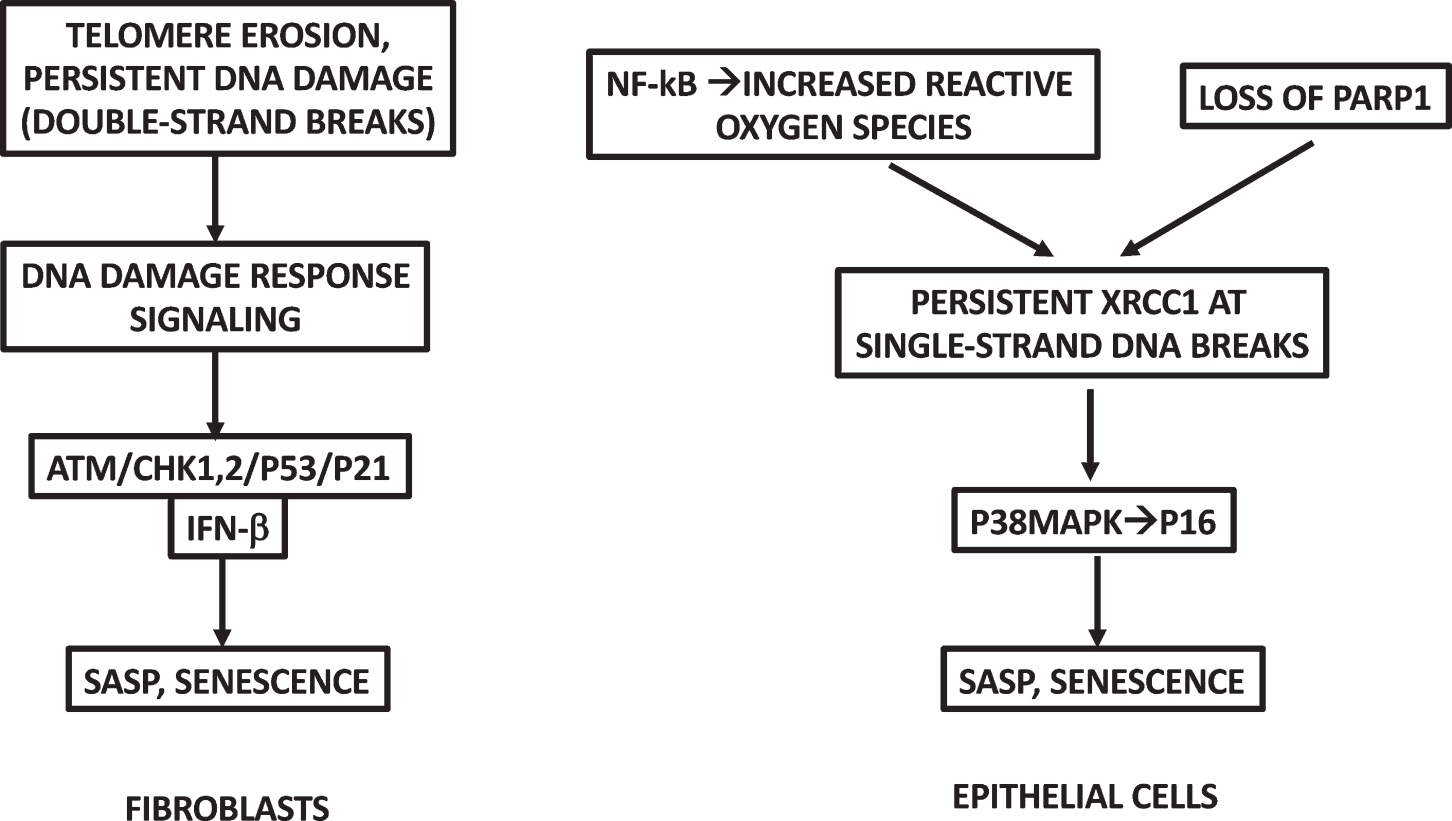
Comparison of DNA damage/IFN‐I/senescence pathways in fibroblasts vs. epithelial cells. See text and (Nassour et al., 2016; Rodier et al., 2009; Romanov et al., 2001) for details. For simplicity, this figure omits independent signaling initiated by cytosolic chromatin fragments through cGAS/STING or other DNA sensors. Note the caveat that, while this study (Nassour et al., 2016) compared matched keratinocytes vs. dermal fibroblasts, it remains possible that other epithelial cell types may exhibit more fibroblast‐like or other DNA damage‐related response pathways. “IFN‐β” in this figure denotes the IFN‐β‐related signaling pathways, not necessarily IFN‐β protein

*Epithelial cells*—the major cell of origin in cancer—exhibit a substantially different, although incompletely understood, senescence program, as first described by the Galloway laboratory in 1998 and the Tlsty laboratory in 2001 (Kiyono et al., 1998; Romanov et al., 2001). An epithelial molecular mechanism was reported by the Abbadie laboratory in 2016 (Nassour et al., 2016). Essentially, epithelial cells undergo a metastable growth arrest at 10‐15 population doublings, accompanied by p16 up‐regulation, but not reflecting senescence. Instead, they downregulate the DNA repair enzyme poly‐ADP ribose polymerase (PARP) and up‐regulate reactive oxygen species (ROS) through an NF‐κB/superoxide dismutase/H_2_O_2_ effect. This ROS generates persistent single‐strand DNA breaks whose repair is suppressed due to PARP deficiency. Interestingly, single‐strand break foci containing XRCC1 engage a p38/p16 pathway, rather than fibroblastic DDR/p53/p21 (and telomere erosion) pathways. These single‐strand breaks are also highly mutagenic. Rarely, but consistently, proliferating subclones arise by spontaneous oncogenic transformation involving epithelial–mesenchymal transition (EMT) (Nassour et al., 2016). Intriguingly, EMT‐driving transcription factors were previously shown to allow senescence bypass during oncogenic transformation by repressing the CDKN2A locus, encoding p16 and p14ARF (Valsesia‐Wittmann et al., 2004), perhaps playing a role in this epithelial senescence bypass. Additionally, EMT was previously shown to decrease cellular H_2_O_2_, perhaps contributing to bypass (Farris et al., 2016). Salient differences in senescence pathways between fibroblasts vs. epithelial cells are depicted in Figure 1. Note the caveat that, while this study compared matched keratinocytes vs. dermal fibroblasts, it remains possible that other epithelial cell types may exhibit more fibroblast‐like or other DNA damage‐related response pathways.

That spontaneous senescence, at least in fibroblasts, is due to telomere erosion, acting through DDR, has generally proven to be true, although the evidence is more direct in some instances than others (reviewed in (He & Sharpless, 2017; Hernandez‐Segura, Nehme, & Demaria, 2018)). Nevertheless, DDR‐induced senescence has been demonstrated abundantly in cancer cells undergoing chemotherapy and radiotherapy (T. Li & Chen, 2018). Moreover, a well‐characterized pathway termed oncogene‐induced senescence (OIS) generally prohibits a single oncogene (e.g., activated Ras) from transforming primary cells, in the absence of complementary gene mutations. This response is thought to represent a specialized form of DNA damage signaling arising from replicative stress in response to mitogenic overstimulation (Bartkova et al., 2006; Di Micco et al., 2006). DNA damage signaling is also thought to promote stress responses, growth arrest, and senescence with respect to other stimuli such as oxidative stress and telomere erosion (Fridman & Tainsky, 2008; Ogrodnik, Salmonowicz, & Gladyshev, 2019; Rudolph et al., 1999). More recently, cytosolic chromatin fragments resulting from dysregulated nuclear lamin B1 protein, emergence of micronuclei, incomplete DNA repair, or down‐regulation of cytoplasmic DNases have been shown to generate a senescent phenotype as well, by novel mechanisms that will be addressed below (Barascu et al., 2012; Dou et al., 2017; Gluck et al., 2017; Graziano, Kreienkamp, Coll‐Bonfill, & Gonzalo, 2018; Takahashi et al., 2018). Nevertheless, other factors that can cause senescence probably function independently of DNA damage, such as unfolded protein responses where proteostasis is defective, and mitochondrial defects; recent recommended reviews on cell senescence have been published (Childs, Durik, Baker, & van Deursen, 2015; Gorgoulis et al., 2019; He & Sharpless, 2017; Hernandez‐Segura et al., 2018; McHugh & Gil, 2018).

## 3 IFN‐I, DNA DAMAGE SIGNALING, AND SENESCENCE

### 3.1 Early observations

Pioneering studies provided the cornerstones for the subsequent elucidation of the critical roles of IFN‐I in DNA damage signaling and senescence. In 1995, the up‐regulation of interferon‐inducible genes, suppressed by IFN‐β‐neutralizing antibodies, was reported in senescent fibroblasts (Tahara et al., 1995). In 2003, Michael Tainsky's laboratory reported the use of oligonucleotide microarrays to identify genes regulated during the pre‐immortalization vs. post‐immortalization vs. senescent fibroblasts, using DNA‐demethylating agents to induce senescence in otherwise immortalized (Li‐Fraumeni) fibroblasts. Interestingly, of the genes that were down‐regulated by DNA methylation in immortalized cells, and up‐regulated in demethylated/senescent cells, 46% were interferons or interferon pathway genes (Kulaeva et al., 2003). Note that this study reported the effect of DNA demethylation in p53‐null cells and that p53 is important for growth arrest to initiate senescence, and p53 was later found not be required for the induction of the SASP, a feature of stable senescence (Coppe et al., 2008), implicating interferon responses in this maintenance phase. This study, together with earlier observations of up‐regulated IFN‐I signaling in senescent cells, implicated interferon signaling in senescence (Fridman & Tainsky, 2008; Tahara et al., 1995).

Also in 2003, the laboratory of Tadatsugu Taniguchi reported that IFN‐I induced p53 expression and synergized with stress stimuli to activate p53 in response to DNA damage (Takaoka et al., 2003). Gerardo Ferbeyre's laboratory showed that prolonged IFN‐β treatment induced cell senescence in fibroblasts, accompanied by DDR features including phosphorylation of ATM, CHK2, and p53, γ‐H2AX foci (indicative of unrepaired double‐strand DNA breaks), and increased ROS (Moiseeva et al., 2006). The senescence was ATM‐ and ROS‐dependent. While the mechanism of how exactly IFN signaling triggered these effects (NF‐kB‐mediated ROS generation, as noted in the previous section?), it provided further support that IFN‐β could induce DDR‐dependent senescence.

It should be noted that IFN‐β, like most SASP components, is not universally expressed in senescence. There are sound reasons to expect this. These include a. the temporal switches from “early” to “late” SASP subprograms noted in multiple studies, b. the existence of IFN‐independent, albeit IRF3‐dependent induction of an important subset of interferon‐inducible genes, which is consistent with the identification of multiple interferon‐inducible cytokines but not IFN‐β itself in the recently published database of the SASP proteome, and c. the potential suppression of IFN‐β by co‐activated signaling, such as p38 MAPK (Ashley et al., 2019; Basisty et al., 2020; Childs et al., 2017; De Cecco et al., 2019; Dou et al., 2017). Nevertheless, IFN‐β and related pathways were functionally identified in the reports discussed here and in a comprehensive gene expression profiling of fibroblasts undergoing replicative and induced senescence (Purcell, Kruger, & Tainsky, 2014). The early reports mentioned here opened a floodgate of subsequent work, showing that DNA damage signaling functions critically through IFN‐I signaling to engage cell senescence.

## 4 DNA DAMAGE/GENOME INSTABILITY CONTRIBUTES TO CELL SENESCENCE VIA IFN‐I

This concept was foreshadowed by transcriptomic observations of IFN‐I pathway signatures in cells and tumors with chemo‐/radiotherapy‐induced DNA damage, which correlated positively with therapeutic response (Burnette et al., 2011; Deng et al., 2014; Minn, 2015; Sistigu et al., 2014; Yu, Katlinskaya, et al., 2015).

A breakthrough was reported in 2015 by Serge Fuchs' laboratory, with rich mechanistic and translational impact (Yu, Katlinskaya, et al., 2015). First, they used inducible, targeted FokI nuclease fusion proteins to produce double‐strand breaks, resulting in IFN‐β IRF7, and phospho‐STAT1 induction. The IFN‐β induction was ATM‐, IKKα/β‐, and IRF3‐dependent; IRF3, surprisingly, localized on chromatin repair foci. Fibroblasts from progeria patients or from Terc‐deficient mice, which senesce prematurely in culture, showed elevated DNA repair foci and IFN‐β expression. Remarkably, neutralization of IFN‐β partially abrogated senescence—as well as in the progeria and normal senescent fibroblasts. They then demonstrated that stem cell defects in mice with short telomeres (Terc^−/−^), manifested in disrupted intestinal crypt‐and‐villus architecture, were partially rescued by knockout of the IFNAR1 receptor, as were markers of intestinal stem cell identity. These results showed that IFN‐I signaling contributed to stem cell senescence. Consistent with previous reports (see above), IFN‐I also contributed to DDR, because the IFNAR1 knockouts had decreased p53BP1‐positive foci, p53 activation, and the senescence markers, p16^INK4a^ and p21^WAF/CIP^. Finally, Terc^−/−^ mice, after breeding for several generations, exhibit a premature aging phenotype. Remarkably, the knockout of the IFNAR1 receptor alleviated this phenotype and enhanced longevity significantly. Overall, this report both elegantly confirmed that IFN‐I induces DNA damage/DDR‐induced senescence in vivo and demonstrated that DNA damage induces IFN‐I.

The degradative loss of membrane‐bound IFNAR1 and suppression of IFN‐I signaling are a common event in human melanoma progression (Araya & Goldszmid, 2017; Fuchs, 2013). Melanocytes undergo oncogene‐induced senescence (OIS, discussed above) in response to activated *Braf* (Campisi & d'Adda di Fagagna, 2007). The Fuchs laboratory showed that the knockout of the IFNAR1 receptor abrogated oncogene‐induced senescence (OIS) conferred by activated *Braf* expression in culture. Consequently, IFNAR1 knockout mice showed accelerated melanoma development after induction of a conditional Braf^V600E^ allele. Conversely, the expression of a stable IFNAR1 mutant in melanoma cells abrogated melanoma metastasis, with tumor cells showing increased IFN‐I signaling and senescence markers. Finally, the therapeutic response to immune checkpoint inhibitors was enhanced in tumors with active IFN‐I signaling. These results provide clear evidence that IFN‐I signaling plays a critical role in OIS—senescence linked to replicative stress/DDR—in vivo.

The mechanism by which IFN‐I fundamentally enhances DNA damage and DDR is not yet understood. Clues may potentially be found in earlier reports, including (a) IRF5 or IRF7 over‐expression can induce DDR‐dependent senescence (Li et al., 2008), albeit through mechanisms not yet understood; (b) IFN‐I increases cellular ROS, potentially damaging DNA (Moiseeva et al., 2006; Takaoka et al., 2003). In this connection, IFN‐γ also induced ROS‐/DDR‐dependent senescence in endothelial cells (Kim, Kang, Seu, Baek, & Kim, 2009); (c) As mentioned above, the persistent expression of (ubiquitinated) IRF3 can induce apoptosis (Chattopadhyay et al., 2016), so, perhaps transient Ub‐IRF3 expression can activate a sub‐apoptotic level of caspase‐dependent nucleases, causing persistent DNA damage; (d) the IFN‐I‐induced cytosolic DNA sensor IFI16 activates ATM‐p53 DDR signaling and inhibits telomerase activity, perhaps contributing to genomic instability (Choubey & Panchanathan, 2016). In this connection, persistent IFN‐I exposure caused telomere loss in T cells (O'Bryan et al., 2011).

Speculatively, IFN‐I could also enhance DNA damage signaling, at least by exogenous stimuli, through a novel indirect mechanism, with important ramifications for IFN‐I bioactivities in general. EMT‐driving transcription factors such as Slug/Snail‐2 and ZEB1 stimulate DNA repair—resolving DDR—through established mechanisms, conferring chemo‐ and radio‐resistance (Gross et al., 2019; Marie‐Egyptienne, Lohse, & Hill, 2013; Zhang et al., 2014). Importantly, basal (unstimulated) IFN‐I gene expression signatures were constitutively higher in primary breast cancer cells of epithelial phenotype as compared with cells of mesenchymal/cancer stem cell (CSC) phenotype, derived from the same tumor (Doherty et al., 2017). IFN‐β treatment induced target genes efficiently in both. Remarkably, IFN‐β treatment also induced substantial mesenchymal‐to‐epithelial transition (MET) and loss of CSC‐related phenotypes. In this connection, an earlier report showed that IFN‐β suppressed TGF‐β‐induced EMT because phosphorylated IRF3 inhibited Smad factors (Xu et al., 2014). As EMT‐driving transcription factors help bypass senescence (see above), this suppression of EMT/induction of MET could provide an indirect pathway to increased senescence.

**FIGURE 2:**
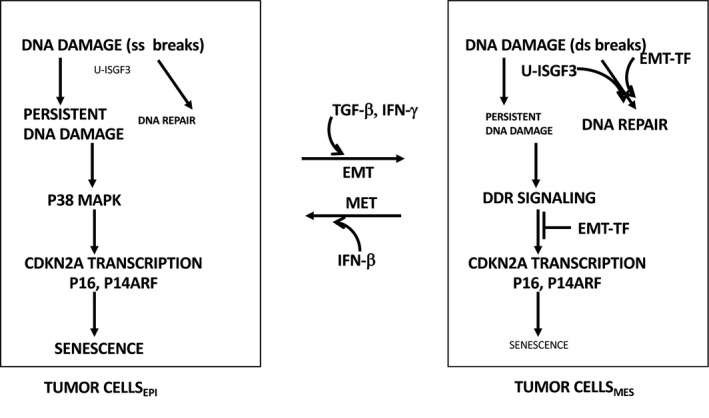
Mechanisms by which epithelial tumor cells enforce senescence more efficiently than tumor cells that have undergone EMT. For simplicity, only selected stimuli of EMT or MET are shown. There are many others

Another molecular detail is notable here. The transcription factor complex ISGF3 is acutely phosphorylated in response to IFNAR1 stimulation by IFN‐I, but an unphosphorylated form of ISGF3 (U‐ISGF3) tends to persist in cells at longer time points after stimulation, due to up‐regulation of its three components (IRF9, STAT1, and STAT2). U‐ISGF3 is highly protective against DNA damage‐induced cell death (Cheon et al., 2013). Accompanying MET, IFN‐β exposure also suppressed U‐ISGF3 expression (Doherty et al., 2017). Thus, IFN‐β treatment could sensitize cells to the cytotoxic effects and pro‐senescent effects of DNA damage, providing a novel interpretation of the results reported above from the Fuchs laboratory. The interplay between EMT/MET and IFN‐I‐related cell senescence is depicted in Figure 2.

The mechanisms by which DNA damage—broadly defined—reciprocally stimulates IFN‐I signaling and cell senescence are emerging. The cGAS‐STING cytosolic DNA receptor has become, legitimately, the center of attention, because of its critical role in innate immunity, senescence, cancer immunotherapy, and ability to sense pleomorphic genomic abnormalities (Gluck & Ablasser, 2019; Khoo & Chen, 2018; Mackenzie et al., 2017; Yang, Wang, et al., 2017; Zhu et al., 2019).

The laboratory of Shelley Berger comprehensively showed the critical role of the cGAS/STING sensor in both senescence and inflammation (Dou et al., 2017). Cytosolic chromatin fragments (CCFs) are observed in senescent cells, apparently due to the down‐regulation and/or disorganization of nuclear lamin B, a major contributor to genomic instability and senescence (Dou et al., 2015; Graziano et al., 2018). Senescence, whether spontaneous or induced, was accompanied, obligatorily, by cGAS/STING activation, which, in turn, activated NF‐kB and SASP genes. The SASP induction was suppressed by lamin B1 over‐expression and, as observed before (Rodier et al., 2009), did not appear to involve p53. RAS^V12^ expression vectors were injected into the livers of wild‐type vs. STING‐knockout mice, revealing that SASP expression and immune rejection of tumor cells were STING‐dependent. This association of CCF‐activated STING with inflammatory gene expression (IL‐1A/B, IL‐6, IL‐8) was supported by clinical tumor expression data. These results demonstrated that genomic instability generates CCFs, activating cGAS/STING, and SASP expression. This study did not directly implicate IFN‐β induction in response to STING activation. Although this was attributed to suppression of the IFN‐β pathway by p38 MAPK, cGAS can also signal to induce the SASP through an interferon‐independent, Toll‐like receptor‐2/acute phase serum amyloid pathway (Hari et al., 2019). Nevertheless, other studies have shown an important role of IFN‐I, in which STING was activated by micronuclei‐derived CCFs or by the RNA‐DNA hybrids that accumulate in RNaseH2‐deficient cells, indicating context dependence of cGAS‐STING‐initiated senescence pathways. (Gentili et al., 2019; Gluck & Ablasser, 2019; Mackenzie et al., 2016, 2017). Alternatively, a subset of “interferon‐inducible genes” is induced in an IRF3‐dependent but IFN‐β‐independent manner, perhaps obviating the need for IFN‐β per se in some contexts (Ashley et al., 2019).

Cyclic GMP‐AMP synthase is considered, by definition, a cytosolic DNA sensor, which recognizes CCFs via a topoisomerase I‐HMG2‐cGAS complex to induce senescence (Zhao et al., 2020). Recent observations show a nuclear‐localized function as well (Gentili et al., 2019). Interestingly, nuclear cGAS localizes preferentially to centromeres and LINE‐1 retrotransposons, which play an important role in IFN‐I‐induced senescence, discussed below.

It should be noted, however, that by no means are all forms of DNA damage sensed by STING; in fact, the DNA damage‐induced/IFN‐β‐dependent senescence reported by the Fuchs laboratory (see above) was STING‐independent. An additional, perhaps complementary role for the IFN‐I‐inducible DNA sensor IFI16 has been proposed (Choubey & Panchanathan, 2016). Broadly, there may prove to be two non‐exclusive pathways that connect DNA damage with IFN‐I and/or senescence: (a) cytosolic chromatin fragments, derived, for example, from micronuclei, sensed by cGAS/STING, and/or (b) persistent DNA repair foci in the nucleus that could, in principle, be sensed by one or more of its components (Dantuma & Pfeiffer, 2016). With regard to the latter, the IFN‐α‐induced tumor suppressor protein, promyelocytic leukemia protein (PML), localizes to both single‐strand and double‐strand DNA repair foci. PML plays a critical role in OIS and spontaneous senescence, suggesting that it might represent one component of the DNA damage/senescence axis (Fu et al., 2015).

Intriguingly, a third pathway of “DNA damage signaling,” broadly defined, may also be envisioned. It has generally been assumed that telomere erosion induces DDR and/or STING signaling through genome‐wide instability. In fact, telomeric DNA damage is uniquely irreparable, inducing a persistent DDR response (Fumagalli et al., 2012). A more direct mechanism has been proposed, however. Frequently, immortalized tumor cells have shorter telomeres than corresponding normal cell types. Longer telomeres corresponded to higher levels of the telomere‐associated RNA, TERRA. Intriguingly, TERRA down‐regulated a subset of IFN‐I target genes, including STAT1, ISG15, and OAS3. (Okamoto & Seimiya, 2019), (Hirashima & Seimiya, 2015). The authors propose that the telomere^short^/TERRA^low^/IFN‐I‐target^high^ phenotype is advantageous for cancer cells. While no specific advantage was proposed, this could hypothetically derive from the immunosuppressive effect of chronic IFN‐I stimulation, mentioned earlier.

**FIGURE 3:**
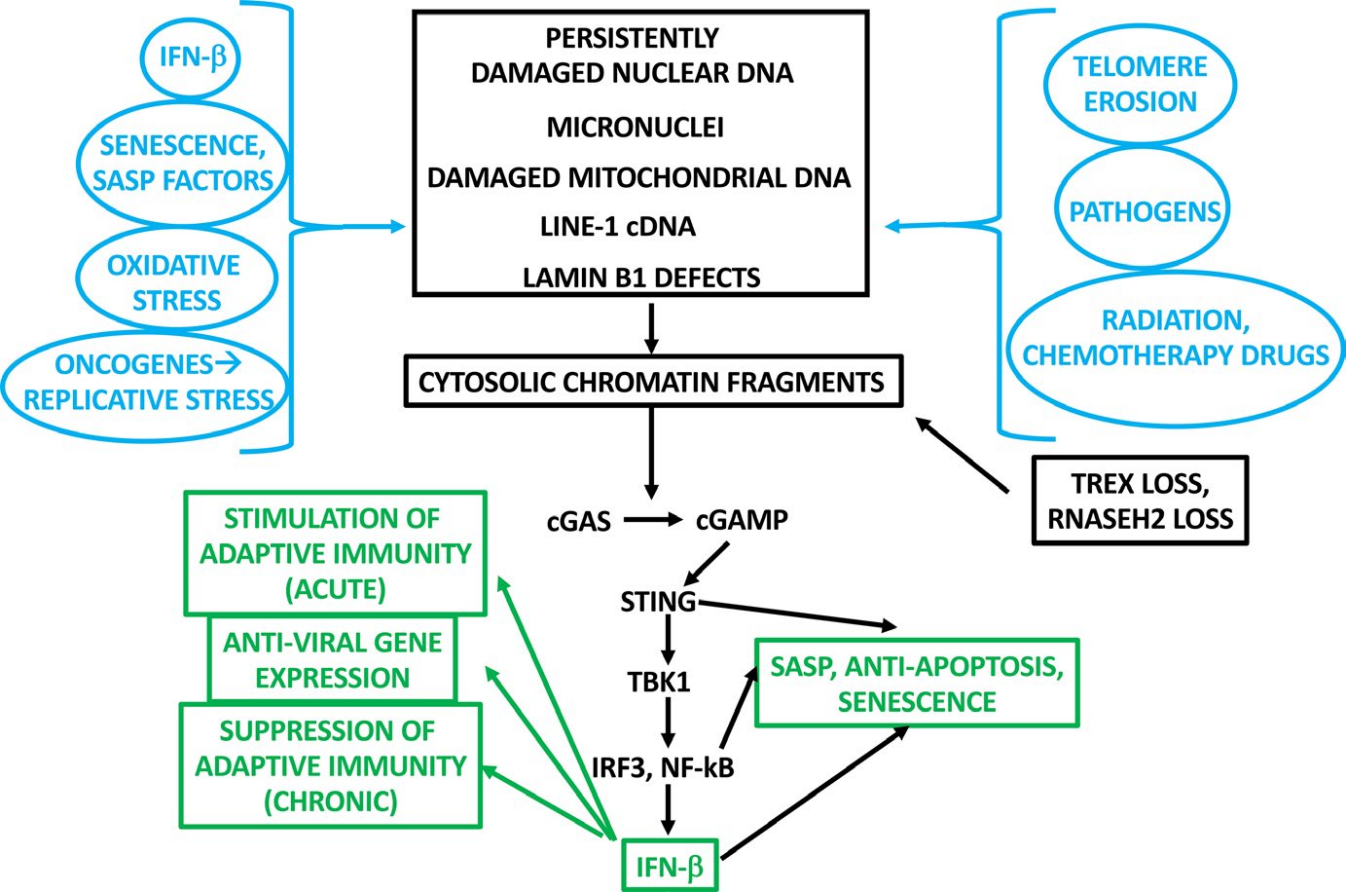
The complex interplay among senescence stimuli, cGAS/STING signaling and downstream effects, both dependent on and independent from IFN‐I. For simplicity, this figure omits conventional DNA damage signaling initiated by ATM and p53 at DNA repair foci in the nucleus. “IFN‐β” in this figure denotes the IFN‐β‐related signaling pathways, not necessarily IFN‐β protein

The complex interplay between DNA damage signaling, cell senescence, and IFN‐I is depicted in Figure 3.

## 5 RETROTRANSPOSONS, IFN‐I, AND CELL SENESCENCE

Retrotransposons, whose transposition is through an RNA intermediate, arise from residual fragments of integrated viral genomes that contribute significantly to genomic instability. While transposons collectively comprise about half of the human genome, fortunately, only one subclass of retrotransposons, long interspersed element‐1 (LINE‐1), has retained reverse transcriptase activity and mobility, which can mobilize both LINE‐1 and other retrotransposons, such as Alu‐1 elements and certain human endogenous retroviruses (HERVs), in trans. Transposition can alter the genome at deletion or integration sites, contributing to genetic disease, aging, and cancer (Burns, 2017; Cardelli, 2018; Maxwell, 2016). Hypomethylation of the LINE‐1 promoter CpG islands in cancer (especially p53‐deficient) and aging promotes transcription and transposition (Burns, 2017; Cardelli, 2018). They are also activated by DNA damage and suppressed in the germline by the PIWI complex and elsewhere by certain interferon‐inducible genes, including MOV10 helicase, APOBEC, and RNaseH2 (Cardelli, 2018). Senescence‐associated chromatin reorganization (perhaps lamin B‐mediated: see above) has been proposed to activate LINE‐1 transcription and transposition (Sedivy et al., 2013).

Transposition is proposed to promote cell senescence and aging, in part, through an important side effect: DNA damage and DDR signaling (Lenart, Novak, & Bienertova‐Vasku, 2018; Sedivy et al., 2013; Toth, Pezic, Stuwe, & Webster, 2016). For example, DNA repair foci are over‐represented at Alu elements in senescent mesenchymal stem cells, and knockdown of Alu transcripts partially reverses senescence (Wang et al., 2011). Additional mechanisms involving other pattern recognition receptors coupled to IFN‐I are described below.

Recent studies have implicated the detection of LINE‐1 DNA‐RNA hybrids in IFN‐I‐mediated cell senescence. Human fibroblasts that undergoing spontaneous, oncogene‐induced, or oxidative stress‐induced senescence up‐regulated LINE‐1 RNA transcripts. These induced IFN‐α/β family genes, in a reverse transcriptase‐dependent and STING‐dependent manner (De Cecco et al., 2019). LINE‐1 up‐regulation was attributed to the loss of the exonuclease Trex, the loss of the LINE‐1 repressor Rb1, and the up‐regulation of FOXA1, a LINE‐1 activator that binds the 5′ UTR. Ablation of IFN expression suppressed the expression of certain SASP components (CCL‐2, IL‐6, and MMP‐3) but not IL‐1β. Both LINE‐1 and IFN‐β expression occurred in a previously overlooked “late‐senescent phase,” subsequent to the phases where DNA damage signaling and SASP induction occurred, again underscoring the temporal switches that characterize the senescence response. Remarkably, treatment of aged mice—which showed elevated LINE‐1 transcripts in certain tissues—with a reverse transcriptase inhibitor decreased IFN‐I and IFN‐I target gene expression, SASP expression, and reversed certain age‐related pathologies such as macrophage infiltration and skeletal muscle atrophy. These results demonstrated that the induction of LINE‐1 cDNA stabilizes certain features of cell senescence through a STING/IFN‐I pathway. Bearing in mind that nuclear cGAS localizes preferentially to LINE‐1 elements, an additional contribution of cGAS as a “transposition sensor” might also be suspected (Gentili et al., 2019).

In a related study from Serge Fuchs' laboratory, mouse embryo fibroblasts were transfected with LINE‐1 expression constructs, resulting in IFN‐β induction that was dependent upon ORF2 endonuclease activity, a component of the LINE‐1 transposition machinery (Yu, Carbone, et al., 2015). IFN‐β induction was therefore proposed to require LINE‐1 transposition, although the potential contribution of DNA breaks themselves, and consequent DDR signaling, was not ruled out. Interestingly, either induced IFN‐β or exogenous IFN‐β inhibited LINE‐1 transposition, by up‐regulating the IFN‐inducible RNA helicase MOV10, a germline transposition suppressor; the knockdown of IFNAR1, conversely, enhanced transposition. This study demonstrated clearly that IFN‐β induced by LINE‐1 transposition and/or LINE‐1 endonuclease activity, is a suppressor of LINE‐1 transposition, forming a negative feedback loop. This implies that LINE‐1 transposition continually contributes to senescence, in part, by inducing IFN‐β, which stabilizes the senescent state while also suppressing LINE‐1 activity.

SIRT6 knockout mice show a progeria phenotype accompanied by sterile inflammation, an excess of cell senescence markers, stem cell exhaustion, and LINE‐1 transcription, as SIRT6 normally represses the latter (Van Meter et al., 2014; Mostoslavsky et al., 2006). These phenotypes were found to be dependent upon LINE‐1 reverse transcription and cGAS‐induced IFN‐I expression, confirming the important contribution of IFN‐I to LINE‐1‐induced senescence and aging (Simon et al., 2019). It is interesting to note that PML protein (see above) is another potent suppressor of retrotransposition (Dutrieux et al., 2015).

In contrast to this effect of IFN‐β, another report demonstrated that IFN‐γ stimulated bidirectional *transcription* (not transposition) of human endogenous retroviruses (HERV), through STAT1, to generate double‐stranded RNAs termed SPARCS (stimulated 3′ antisense retroviral coding sequences) (Canadas et al., 2018). This, and reverse‐transcribed cDNA, activated MAVS and cGAS/STING to induce IFN‐β. While increasing immune infiltration, the net effect was immunosuppressive (e.g., PD‐L1 was also induced), consistent with the dual nature of interferon effects on immunity noted previously (Lee & Ashkar, 2018; Lukhele et al., 2019; Snell et al., 2017). Increased mesenchymal gene expression was noted in tumors with elevated HERV expression, suggesting that IFN‐γ‐induced EMT, (Lo et al., 2019) bypassing epithelial‐type senescence (discussed above).

Treatment of ovarian cancer cell lines with the DNA demethylation agent 5‐aza‐2'deoxycytidine, or breast cancer cells with inhibitors of the histone demethylase LSD1, also induced double‐strand RNA transcription from HERV sequences, activating IFN‐β and immune surveillance, with important clinical implications, likely including tumor cell senescence (Chiappinelli et al., 2015; Sheng et al., 2018; Stone et al., 2017). This remains to be tested, however.

Finally, age‐dependent changes in DNA methylation signatures, “DNAm PhenoAge,” have proven to correlate with chronologic age and to predict longevity reliably (Levine et al., 2018). Methylation phenotypes associated with aging also associate with an IFN‐I/pro‐inflammatory gene expression pattern. Global chromatin reorganization accompanies cell senescence, driven by lamin B1 defects (discussed above) as well as altered H3 K9 methylation (Chandra et al., 2015). Interestingly, H3 K9 methylation is, in some contexts, a pre‐requisite for DNA repair and site‐specific DNA methylation, and decreased H3 K9 methylation triggers SASP expression, raising the possibility that altered histone methylation may drive the senescent inflammatory phenotype, via altered DNA methylation (Du, Johnson, Jacobsen, & Patel, 2015; Hernandez‐Segura et al., 2018; Takahashi et al., 2012).

## 6 FUTURE PERSPECTIVES

- Cell senescence in fibroblasts is engaged via telomere erosion and persistent DNA damage signaling and stabilized via the induction of epigenetic noise as well as the SASP. SASP induction was shown to context dependently involve either IFN‐I induction, or, at least, pattern recognition receptors, such as cGAS/STING, a component of the innate immune response. It is not known what role, if any, IFN‐I plays either upstream or downstream of the epigenetic noise that characterizes senescent cells. This remains an important open question.
- Epithelial cell senescence follows a very different path than fibroblasts, showing that cell identity is a major factor in senescence. Unfortunately, the role of interferons in epithelial cell senescence has not yet been reported. This would be crucial for understanding interferons' role in normal development and homeostasis, as well as disease processes, especially cancer, which is derived mainly from epithelial cells. In this light, it should be noted that epithelial vs. mesenchymal phenotypes are not alone in controlling interferon responses through the cellular differentiation state. In fact, human embryonic stem cells are uniquely non‐responsive to exogenous IFN‐I; instead, they constitutively express a subset of interferon‐stimulated genes to maintain an antiviral response, which shifts to conventional, inducible expression upon differentiation (Wu et al., 2018). An understanding of how cellular differentiation states impact upon senescence through IFN‐I or related pathways is another important goal.
- In the context of cancer, EMT‐driving transcription factors overcome oncogene‐induced senescence, an effect termed “cellular pliancy” (Puisieux, Pommier, Morel, & Lavial, 2018). Does EMT affect IFN‐I expression or function, and, if so, is this an underlying cellular pliancy mechanism? In this connection, EMT suppressed the expression of IFN‐I and IFN‐III in human airway epithelial cells, through down‐regulation of IRF1 (Yang, Tian, et al., 2017).
- The mechanism by which IFN‐I induces DNA damage and DDR signaling is understood incompletely. For example, does IFN‐I induce cytosolic chromatin fragments? DNA‐SCARS?
- Chronic inflammation leading to senescence and aging phenotypes, with increased susceptibility to age‐related disease, has been termed “inflammaging” (Fulop, Witkowski, Olivieri, & Larbi, 2018). The role of IFN‐I in inflammaging remains to be investigated.
- IFN‐I has been implicated not only in senescence, but also in aging, for example, in the Terc^−/−^ mouse model cited above, and with regard to loss of brain function/cognitive decline, via IFN‐I signaling in the choroid plexus (Baruch et al., 2014). What is the larger significance of IFN‐I in aging?

## CONFLICT OF INTEREST

There is no conflict of interest to declare.
